# Case report: Neurofibromatosis type 1 gastrointestinal stromal tumor and small bowel adenocarcinoma with a novel germline *NF1* frameshift mutation

**DOI:** 10.3389/fonc.2022.1052799

**Published:** 2022-12-22

**Authors:** Wuming Zhang, Xianqin Hu, Zhikang Chen, Chen Lai

**Affiliations:** ^1^ Department of General Surgery, Xiangya Hospital of Central South University, Changsha, Hunan, China; ^2^ Hunan Key Laboratory of Precise Diagnosis and Treatment of Gastrointestinal Tumor, Xiangya Hospital of Central South University, Changsha, Hunan, China; ^3^ International Joint Research Center of Minimally Invasive Endoscopic Technology Equipment & Standardization, Xiangya Hospital of Central South University, Changsha, Hunan, China

**Keywords:** NF1 mutation, NF-1 = neurofibromatosis type 1, small bowel adenocarcinoma, GIST - gastrointestinal stromal tumor, PMS2 gene

## Abstract

A synchronous case of small bowel adenocarcinoma(SAB) is reported, accompanied with gastrointestinal stromal tumor(GIST),and gangliocytomain in an elderly woman with neurofibromatosis type 1 (NF-1). A 67-year-old female was hospitalized with the chief complaint of abdominal pain, the computed tomography scan indicated a large bowel mass. Multiple tumors were found in the small intestine, through which two larger tumors (7 cm and 1.5 cm) were resected. A novel germline *NF1* mutation and a *PMS2* mutation were identified after genetic testing, followed by the exploration of possible relationship between them in promoting tumorigenesis. Our results suggest multiple gastrointestinal tumors emerging in NF1 patients, and genetic testing can better guide postoperative treatment in a more efficient way.

## Introduction

Neurofibromatosis type 1(NF-1) occurs as an autosomal dominant genetic disease induced by a mutation in the *NF1* gene on chromosome 17 with an incidence of 1 in 3000 (1). The *NF1* gene spans approximately 280kb of genomic DNA, which is composed of 57 constitutive exons and 4 alternate splicing exons (9a, 10a2, 23a, and 48a), serving as a cancer suppressor gene. *NF1* gene known to encode neurofibrin, accompanied with a functional domain, RAS-GAP. This domain plays a role in accelerating the conversion of active RAS-GTP to inactive RAS-GDP, acting as a negative regulator of RAS signaling pathway, which is involved in cell growth and differentiation (2). Neurofibromatosis type 1 is manifested by multiple flat, light-brown patches of skin pigment, skin fold freckling, visible neurofibromas under the skin and Lisch nodules (3).Severe complications, including learning disabilities, scoliosis, osteoporosis and malignancy may present, which can affect all parts of the body, followed by serious impacts on the physical and mental health of patients (4). Only neurofibromas with symptoms requires treatment. Large skin neurofibromas can be removed by surgery. While small and medium-sized neurofibromatosis can be treated with Carbon dioxide laser under anesthesia. The most recent treatments, such as Cabozantinib, is remaining in the experimental stage II (5).

Gastrointestinal stromal tumor (GIST) is a mesenchymal tumor developed from pacemaker cells in the gastrointestinal tract, called Cajal cells, which play an essential role in gastrointestinal peristalsis. Abdominal pain, gastrointestinal obstruction and upper gastrointestinal tract bleeding are presented as the primary manifestations of GIST (6).

Small bowel adenocarcinoma (SBA) occupies 40% of small bowel cancers with a low incidence rate of 7.3 per million people, mainly occurring in the duodenum, jejunum and ileum (7). SBA generally accompanied by local tumor complications. A duodenal SBA is often associated with gastric outlet obstruction, and a jejunal or ileal SBA with abdominal cramps, of which approximately 1/4-1/3 report gastrointestinal bleeding (8). Despite the occupation of 75% of the length and 90% of the mucosal surface of the gastrointestinal tract (9), the incidence of small bowel adenocarcinoma is much decreased compared to colorectal cancer, so most treatments are proposed based on the treatment model of colorectal cancer. This discrepancy may result from a lower incidence of *APC* mutations, where the lower bacterial load, faster food flow and more intestinal juice dilute cause less exposure of harmful material and small intestine has abundant lymph nodes, the higher the IgA levels are correlated with the better immune response (10).

Here, we report a case of multiple primary malignant tumors (NF-1, GIST, SBA and Gangliocytomain), and investigate the possible relationship between them.

## Case presentation

### Clinical evaluation

**Figure 1:**
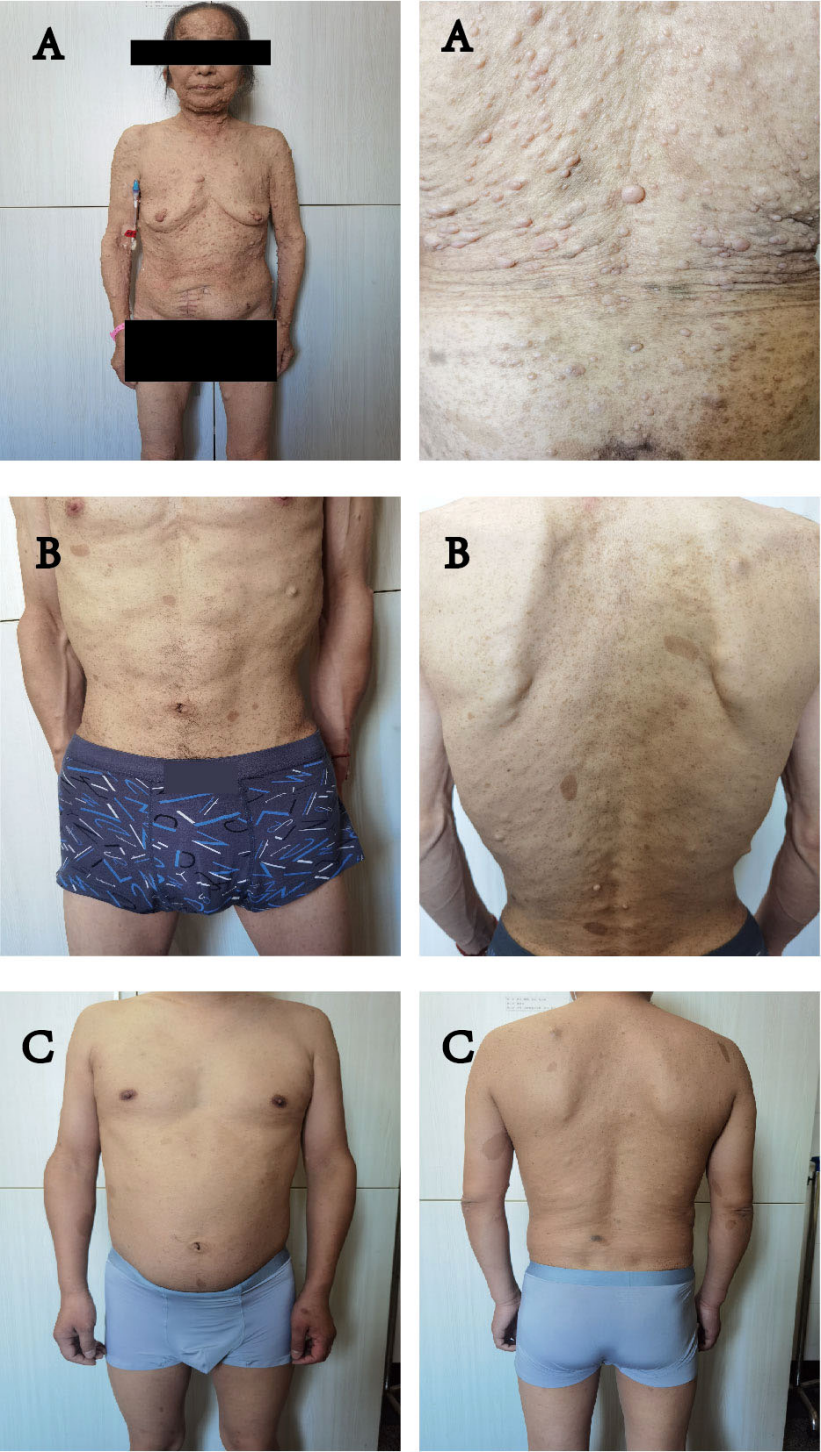
**(A–C)** all showed symptoms of NF1: multiple flat, light-brown patches of skin pigment, skin fold wrecking, visible neurofibromas under the skin. **(A)** is the mother of **(B, C)**.

**Figure 2:**
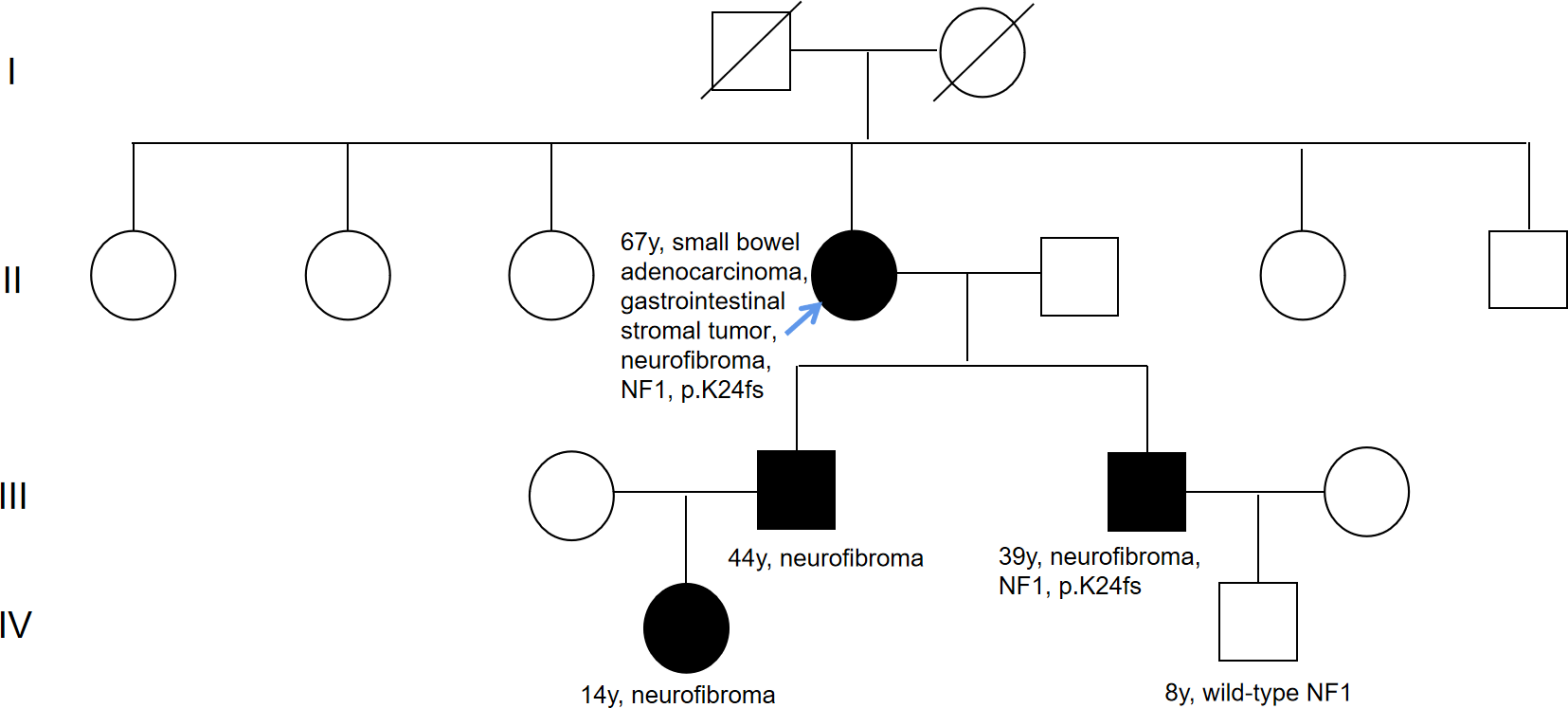
Pedigree of family. The black symbols indicate neurofibromatosis type 1. Numbers on right-top gives the current age. The blue arrow means the proband.

On June 30 2022, a 67-year-old woman was admitted to hospital with the chief complaint of abdominal pain over 2 months. The patient has undergone colonoscopy at another hospital, which revealed multiple circular lumps in the terminal ileum and small polyps in the rectum and colon. On admission, a physical examination was performed, with neurofibroma, café au lait macules, skin fold freckling detected all over the patient’s body (**Figure 1**), which were diagnosed as gastrointestinal neoplasms and neurofibromatosis type 1 (11). A detailed family history survey revealed that the patient’s sons and granddaughter also had neurofibromatosis (**Figure 2**). Improvement of relevant tests: blood routine, urine, feces, coagulation function, tumor markers were normal, fecal latent blood test was positive. Computed tomography for thoracic, abdominal and pelvic examination revealed a terminal ileal tumor with secondary intussusception, diffuse osteoporosis (secondary right second costal old fracture with callus formation), lumps behind the bilateral sacral bone, sacral bone resorption. Multiple subcutaneous nodules were observed on the chest, neck, abdomen and back. (**Supplementary Figure 1**) MRI of head showed no abnormality.

### Surgical findings

On July 04 2022, the exploratory laparotomy was performed under general anesthetic. During the operation, a mass with the size of about 5 cm × 7 cm was found within 10 cm of the ileocecal area, with significant enlargement and edema of the proximal small bowel. A mass approximately 1.5 cm in diameter can be seen on the lateral wall of the small intestine, approximately 120 cm from the Treitz ligament (**Supplementary Figure 2**). The ileocecal tumor was resected, accompanied with its corresponding mesentery, and the mass on the lateral wall of the small intestine.

### Postoperative pathology

Postoperative pathology (**Supplementary Figure 1**) confirmed the mass on the ileocecal area of the small intestine as moderate-differentiated adenocarcinoma, invading the subserosal connective tissues, without metastasis to the mesenteric lymph nodes (0/15). The mass on the lateral wall of small intestine was identified as gastrointestinal stromal tumor, with the maximum diameter of the tumor was 1.5 cm, the tumor envelope was intact, the mitosis count was ≤5/5 mm2, indicating a rather low risk of invasion.

### Postoperative therapy

The proband’s postoperative course went well and the patient was discharged from the hospital one week later. In this case, the metastasis of small bowel adenocarcinoma was not found, also without high-risk features, such as positive resection margins, <8 lymph nodes, tumor perforation, perineural or lymphovascular invasion and poor-differentiated histology. Moreover, the Immunohistochemistry results suggested defective mismatch repair (dMMR). According to NCCN guidelines (8), this case dose not require chemotherapy. In the first three years, it is suggested be observed every three months.

### Gene analysis

**Figure 3:**
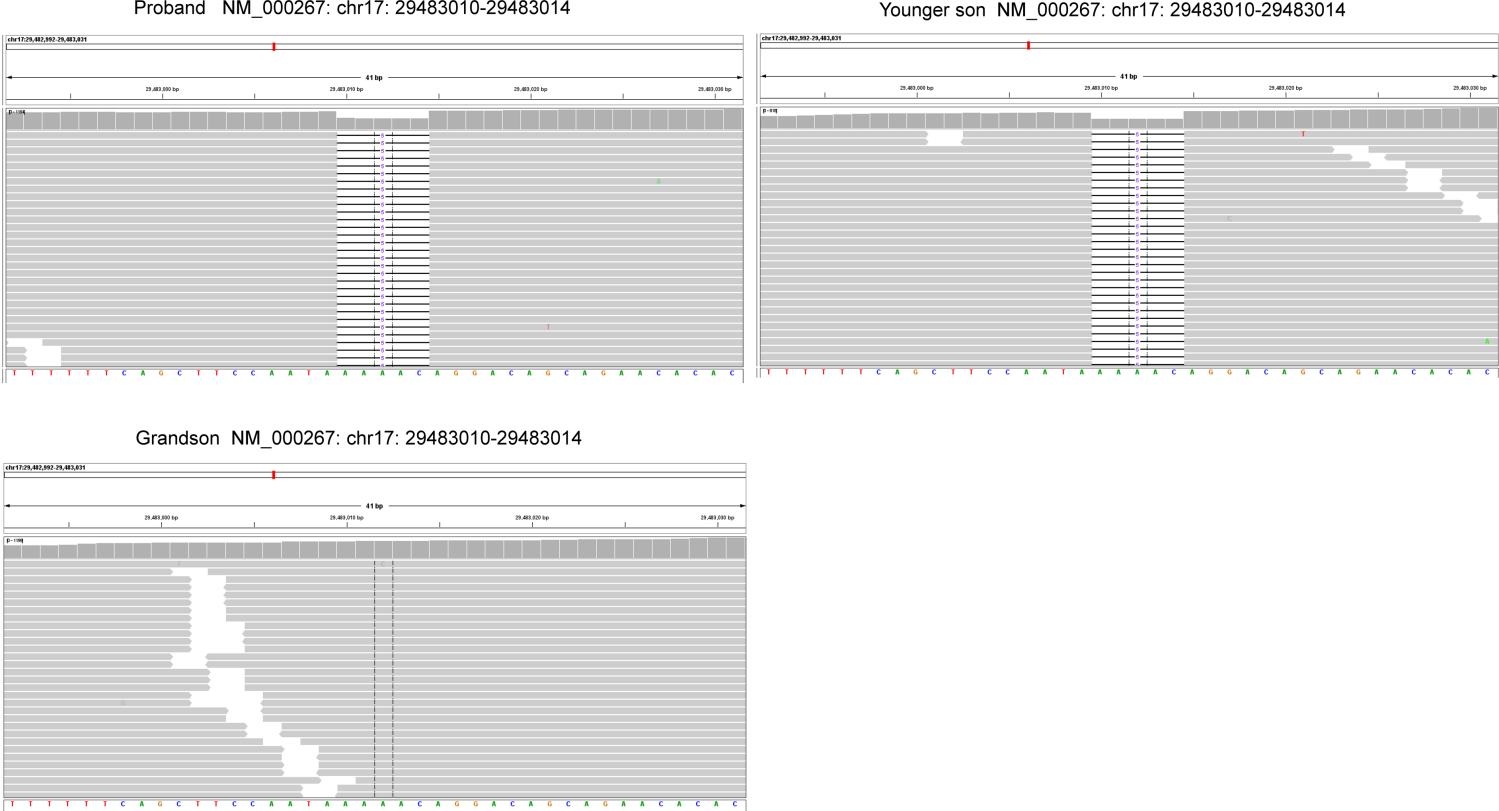
The Integrative Genome Viewer (IGV) snapshot of NF1.

The resected SBA tumor tissues fixed by formalin and embedded with paraffin were subjected to next-generation sequencing (NGS) accompanied with peripheral blood from the proband, with a 1123-gene panel utilized (ChosenMed Technology [Beijing] Co. Ltd, Beijing, China). First, genomic DNA was extracted from the FFPE sections of tumor tissues using AllPrep FFPE DNA/RNA Kit (Qiagen). Pure DNA was eluted in elution buffer. Afterwards, DNA sample library were prepared according to the protocols, which was eventually sequenced with Illumina NextSeq 550Dx (12–14). The next-generation sequencing outcomes revealed a somatic *PIK3CA* mutation (NM_006218: exon10: c.1633G>A: p.E545K), with an allele frequency of 1.43%, followed by, a novel *NF1* germline frameshift mutation (NM_000267: exon2: c.71_75del: p.K24fs),with the variant allele frequency of 45.51%. This *NF1* variation has never been reported in any population database or publications, including the Exome Aggregation Consortium and 1000 Genomes Project. The Integrative Genome Viewer snapshot of *NF1* is depicted in **Figure 3**. In addition, *PMS2* and *PTEN* missense mutations were also detected (**Table 1**). The resected GIST tumor tissues fixed with formalin and embedded with paraffin from the proband were also subjected to next-generation sequencing by the same gene panel. The results revealed no other mutations detected (such as *KIT*, *PDGFRA*) except the same *NF1* frameshift mutation (NM_000267: exon2: c.71_75del: p. K24fs).

**Table 1:** Genetic test results using the resected formalin fixed and paraffin embedded small bowel adenocarcinoma tumor tissues.

Germline mutations							
Gene	Chromosome	Exon	Nucleotide	Amino acid	ClinVarannotation	MutationTaster prediction	SIFT prediction
*NF1*	17	2	c.71_75del	p. K24fs		Disease causing	Disease causing
*PMS2*	7	5	c.379G>A	p. A127T	Conflicting interpretations of pathogenicity		
*PTEN*	10	8	c.949G>A	p. V317I	Uncertain significance		
Somatic mutations							
Gene	Chromosome	Exon	Nucleotide	Amino acid	OncoKB annotation		
*PIK3CA*	3	10	c.1633G>A	p. E545K	Oncogenic		

Considering the impacts of neurofibroma on several family member, gene sequencing was conducted among this family using a hereditary-cancer-gene panel. The results indicted the same *NF1* mutation in the peripheral blood of proband’s younger son, who is 39 years old and diagnosed with neurofibroma. The proband’s grandson detected with wild-type *NF1* (**Figure 3**).

Of note, a germline *NF1* mutation (NM_000267: exon2: c.71_75del: p.K24fs) was detected in the peripheral blood of the proband through next-generation sequencing, which was also detected in one family member affected with NF-1. As the silico analysis with the MutationTaster and SIFT software predicted (15), the *NF1* mutation is disease-causing as the c.71_75del introduced a premature stop codon early in the translation, which resulted in a truncated proteins (**Table 1**). These results indicated that the novel *NF1* mutation might be responsible for the occurrence of NF1 in this family.

## Discussion

Neurofibromatosis usually emerges as a relatively common autosomal dominant disease. Cases of *NF1* combined with stromal tumor have also been occasionally reported, but not cases of NF-1, GIST and SBA.

Neurofibromatosis can be considered a tumor susceptibility syndrome as it brings the increased risk of multiple tumors (16). A study in Finland reported that the cumulative cancer risk of 38% on *NF1* carriers at age 50, and the lifetime cancer risk of 59.6%, which were 3.9% and 30.8% in the general population, respectively. *NF1*-related tumors included malignant peripheral nerve sheath tumor, gastrointestinal adenocarcinoma, GIST, breast cancer, and pheochromocytoma (4).

At present, GISTs are mainly induced by *KIT* gene or *PDGFRA* gene mutations, which are observed in approximately 83% of GIST cases (17). These patients, except for the *PDGFRA-D842V* mutation, exhibit a satisfactory response to imatinib (18). However, *NF1*-related GIST is significantly distinguished from sporadic GIST. First, sporadic GIST is mainly distributed in the stomach (50%-70%), while *NF1*-related GIST is mostly found in the small bowel (68%); Most *NF1*-related GIST is spindle cell type (75%), while the prognosis is better than sporadic GIST (19). Secondly, *KIT* and *PDGFRA* mutations are rarely reported in *NF1*-associated GIST. *NF1*-related GIST is a tumor with low malignancy and risk, with resistance to imatinib, which generally do not require treatment (20). There may exist a common pathway between *NF1*-related GIST and sporadic GIST, that is, *NF1* gene mutation destroys the normal function of neurofibromin and activates structural RAS, which increases downstream signaling *via* the mitogen-activated protein kinase (MAPK) pathway (21). The constitutive activation of the RAS-MAPK cascade could also result from mutations of *KIT* (22). Therefore, the activation of the MAPK cascade can be concluded that the activation of this common pathway leads to the development of GIST (23).

Due to a germline mutation in the *PMS2* gene, Lynch syndrome was diagnosed despite no family history of the tumor. GLTen Kate demonstrated the lifetime risk of SBA in Lynch syndrome to be 4.2% (24). Neurofibromatosis, Lynch syndrome and SBA seem to be correlated to each other. In early embryonic development, the *NF1* gene is a mutational target of MMR deficiency, that its inactivation is a critical step in the malignant progression of MMR-deficient ells (25). Homozygous MMR gene mutations (*MLH1, MSH2, PMS2, MSH6* biallelic mutations) are associated with neonatal neurofibromatosis and are more prone to early severe malignancies, including hematological malignancies, lymphomas and gastrointestinal malignancies (26–28).

Small bowel adenocarcinoma is generally characterized by dMMR associated with Lynch syndrome. The largest retrospective study on SBA identified dMMR protein in 26% (26/100) of tumors, which identified 10% (10/100) of Lynch syndrome in SBA, and 38.5% (10/26) of Lynch syndrome in dMMR tumors (29). In the study of Schrock et al., SBA is associated with a higher mutation rate of NF1 compared to gastric cancer (P=0.049) and colorectal cancer (P<0.01) (30). As a tumor suppressor gene, NF1 mutation may be more likely to lead to the occurrence of SBA under some mechanism. In this case, genetic analysis may contribute to further revealing an association between SBA and NF-1.

## Conclusion

We revealed a novel *NF1* germline frameshift mutation, which exerts a certain contribution to analyzing the relationship between NF-1, GIST, and SBA. If there occur gastrointestinal symptoms in the patients with *NF1* mutation, the possibility of a gastrointestinal tumor should be suspected. Genetic testing is also recommended in these patients for formulating effective treatments.

## Data availability statement

The original contributions presented in the study are included in the article/**Supplementary Material**. Further inquiries can be directed to the corresponding author.

## Ethics statement

Written informed consent was obtained from the individual(s) for the publication of any potentially identifiable images or data included in this article.

## Author contributions

XH and ZC contributed to data acquisition and analysis. WZ and CL contributed to study design, data acquisition, analysis and the writing of the manuscript. All authors contributed to the article and approved the submitted version.
