# Supplementary material for: Case report: Neurofibromatosis type 1 gastrointestinal stromal tumor and small bowel adenocarcinoma with a novel germline NF1 frameshift mutation
Source: Front Oncol. 2022 Dec 22;12:1052799. doi: 10.3389/fonc.2022.1052799 (PMC9815498; doi:10.3389/fonc.2022.1052799)
Supplement: Supplementary file 1 [file DataSheet_1.pdf]

Supplementary table.1 Revised diagnostic criteria for neurofibromatosis type 1 (NF1).

A: The diagnostic criteria for NF1 are met in an individual who does not have a parent diagnosed with NF1 if two or more of the following are present:

1. Six or more café-au-lait macules over 5 mm in greatest diameter in prepubertal individuals and over 15 mm in greatest diameter in postpubertal individuals
2. Freckling in the axillary or inguinal region
3. Two or more neurofibromas of any type or one plexiform neurofibroma
4. Optic pathway glioma
5. Two or more iris Lisch nodules identified by slit lamp examination or two or more choroidal abnormalities (CAs)—defined as bright, patchy nodules imaged by optical coherence tomography (OCT)/near-infrared reflectance (NIR) imaging.
6. A distinctive osseous lesion such as sphenoid dysplasia, anterolateral bowing of the tibia, or pseudarthrosis of a long bone.
7. A heterozygous pathogenic NF1 variant with a variant allele fraction of 50% in apparently normal tissue such as white blood cells

B: A child of a parent who meets the diagnostic criteria specified in A merits a diagnosis of NF1 if one or more of the criteria in A are present

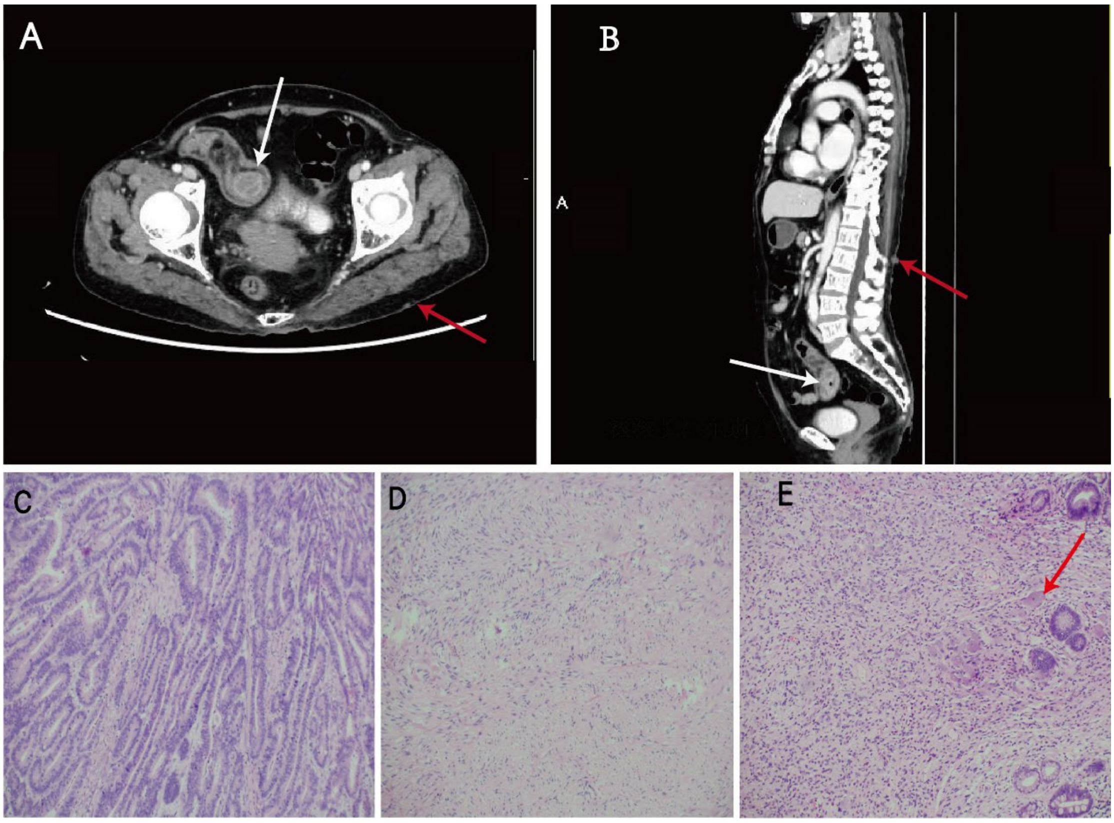

Supplementary Fig.1 CT and pathology：

A and B： Small bowel adenocarcinoma (white arrow). Neurofibromas-soft bumps on or under the skin (red arrow).

C: Small bowel adenocarcinoma. Immunohistochemistry(IHC):Ki-67 （approximately 85%+）, p53（approximately 10%+）, EGFR（approximately 100%+）, HER-2（approximately 35%+）, MSH2 （+）, MSH6(+), EBER(-), PMS2(-), MLH1(-), BRAF V600E(-).

D: Gastrointestinal stromal tumor. IHC:CD117(+), CD34(+), KI67(2%), SDHB(+), S-100(-),SMA(-)

E: Ganglioneuroma. IHC: S-100(+), SOX10(+), NF-Pan(+), CgA(+) and Ki-67（approximately 2%）

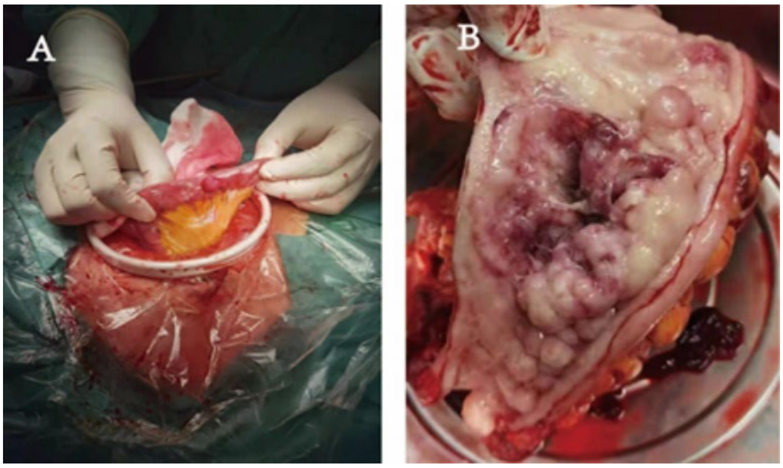

Supplementary Fig.2 Morphology in the surgery.

A: An exophytic gastrointestinal stromal tumor approximately 1.5 cm in size.

B: Ulcerative small bowel adenocarcinoma, approximately 7 cm long, with multiple intestinal neurofibromas.
